# Psychosocial Impairment and Micronutrient Deficiencies in Indian Adolescents: A Pilot Study

**DOI:** 10.7759/cureus.105404

**Published:** 2026-03-17

**Authors:** Afreen Khan, Kanika Yadav, Sangeeta Yadav, Lucky Manik, Sana Alam

**Affiliations:** 1 Pediatrics, Hamdard Institute of Medical Sciences and Research, New Delhi, IND; 2 Biochemistry, Hamdard Institute of Medical Sciences and Research, New Delhi, IND

**Keywords:** adolescent nutrition, child and youth mental health, india, serum ferritin, serum vitamin b12

## Abstract

Background: Adolescent mental health disorders represent a growing concern in India, with potential links to nutritional factors remaining underexplored.

Objective: This pilot study aimed to investigate the association between micronutrient deficiencies (vitamin B12, ferritin, folate, vitamin D, and calcium) and psychosocial impairment (defined as Youth Pediatric Symptom Checklist (Y-PSC) score ≥30) among Indian adolescents attending a tertiary care hospital in Delhi.

Methods: A cross-sectional analytical study was conducted over six months at a tertiary care hospital in Delhi. A total of 96 adolescents aged 11-18 years were enrolled through consecutive sampling. Psychosocial functioning was assessed using the Y-PSC, with scores ≥30 indicating impairment. Venous blood samples were analyzed for hemoglobin, serum ferritin, vitamin B12, folic acid, 25-hydroxyvitamin D, and total calcium levels.

Results: Psychosocial impairment was found in 15.6% (15/96) of participants. Adolescents with psychosocial impairment (n=15) had significantly lower mean serum vitamin B12 levels (191.86±63.00 pg/mL) compared to those without impairment (291.09±207.02 pg/mL), with P=0.039 for vitamin B12. Median serum ferritin levels were significantly lower in the impaired group (28.50 ng/mL, IQR: 21.00-42.00) compared to the non-impaired group (45.00 ng/mL, IQR: 24.00-78.00; Mann-Whitney U test, P=0.041). Vitamin B12 deficiency was present in 80% (12/15) of adolescents with psychosocial impairment versus 23.4% (19/81) in those without impairment (P<0.001). In unadjusted analyses, both vitamin B12 deficiency (OR=13.11; 95% CI: 3.35-51.28; P<0.001) and lower ferritin levels were significantly associated with psychosocial impairment. Multivariable logistic regression analysis identified vitamin B12 as significantly associated with psychosocial impairment (OR=0.985; 95% CI: 0.972-0.997; P=0.017), while the association with ferritin did not reach statistical significance in the adjusted model (OR=0.976; 95% CI: 0.948-1.004; P=0.098).

Conclusions: Preliminary associations were observed between psychosocial impairment and deficiencies in vitamin B12 and ferritin among Indian adolescents in this pilot study. While causality cannot be established from this cross-sectional design, these findings suggest the potential value of incorporating micronutrient screening into routine psychosocial assessments and warrant further investigation in larger, adequately powered prospective studies.

## Introduction

Adolescence represents a critical developmental period characterized by substantial neurobiological, cognitive, and psychosocial changes [1,2]. Despite its potential for positive development, this transitional phase is increasingly associated with rising mental health concerns globally. The World Health Organization reports that one in seven individuals aged 10-19 years experiences a mental disorder, contributing to 13% of the global disease burden in this demographic [3,4].

The etiology of adolescent psychosocial impairment involves complex interactions between biological, environmental, and nutritional factors. Micronutrients, particularly vitamin B12, iron, folate, and zinc, play crucial roles in neurocognitive development and neurotransmitter synthesis [5,6]. However, limited high-quality research has examined their association with mental health outcomes in adolescent populations, particularly in India, where micronutrient deficiencies remain prevalent.

In the context of India's double burden of malnutrition, encompassing both undernutrition and emerging obesity, micronutrient deficiencies may persist even among individuals with normal anthropometric parameters. While the impact of micronutrient status on neurodevelopment is well established in early childhood and cognitive decline in later adulthood, its relevance during adolescence remains underexamined. This pilot study aimed to assess the association between micronutrient deficiencies and psychosocial impairment among Indian adolescents. The primary objective was to investigate the association between serum vitamin B12 level and psychosocial impairment (defined as Youth Pediatric Symptom Checklist (Y-PSC) score≥30) in Indian adolescents. Secondary objectives included exploring associations between psychosocial impairment and other micronutrients (serum ferritin, serum folate, 25-hydroxyvitamin D, and total serum calcium) as well as hemoglobin levels.

It was hypothesized that adolescents with micronutrient deficiencies, particularly vitamin B12 and iron deficiencies, would exhibit higher rates of psychosocial impairment than those with adequate micronutrient status. Given the exploratory pilot nature of this study and evaluation of multiple micronutrients, we acknowledge the increased risk of Type I error and interpret all findings as hypothesis-generating rather than confirmatory.

## Materials and methods

This analytical cross-sectional pilot study was conducted over six months from December 2024 to May 2025 in the Outpatient Department of Pediatrics at Hamdard Institute of Medical Sciences and Research, New Delhi, India. The study protocol received approval from the Institutional Ethics Committee (HIMSR/00251/2024).

Adolescents aged 11-18 years presenting to the pediatric outpatient department during the study period were consecutively approached, and written informed consent was obtained from participants and their guardians. During the clinical interview, information was systematically collected on current medications (including oral contraceptives, anticonvulsants, proton pump inhibitors, and metformin), recent infections or inflammatory conditions, and chronic diseases. Adolescents with known chronic illnesses or those on medications known to affect micronutrient metabolism were excluded from the study to reduce confounding.

As this was designed as a pilot study, a formal a priori power calculation was not performed. The sample size of 96 participants was determined based on feasibility considerations within the six-month study period and available resources. Post hoc power analysis indicated that, with 15 cases of psychosocial impairment (15.6% prevalence) and 81 controls, the study had approximately 70% power to detect an odds ratio (OR) of 3.0 for vitamin B12 deficiency at α=0.05, assuming a baseline prevalence of 25% in the non-impaired group. This modest power underscores the exploratory nature of the study and the need for larger confirmatory investigations.

Participants underwent structured interviews and comprehensive clinical examinations. Data collection included demographic information, dietary intake patterns, physical activity levels, screen time, and socioeconomic background using a prestructured proforma. Dietary assessment was done by the 24-hour recall method and broadly categorized as vegetarian or non-vegetarian based on reported consumption of meat, fish, or eggs.

Socioeconomic status (SES) was evaluated using the Modified Kuppuswamy Scale (2022 update), a validated composite index that incorporates the education level of the head of household, occupation, and monthly family income. Families were classified into upper, upper-middle, lower-middle, upper-lower, and lower socioeconomic classes according to standard scoring criteria.

Physical activity levels were categorized based on WHO guidelines [7] into sedentary, moderate, and vigorous intensity. Specifically, adolescents were classified as: (1) sedentary if they engaged in <60 minutes per day of moderate-to-vigorous physical activity (MVPA); (2) moderate if they engaged in 60-150 minutes per week of MVPA; and (3) vigorous if they engaged in >150 minutes per week of MVPA. Physical activity was assessed through structured interview questions about typical daily activities, participation in sports, active transportation (walking/cycling to school), and recreational activities, following WHO recommendations for adolescent physical activity assessment [8].

Anthropometric assessments were performed using standard protocols. Height was measured to the nearest 0.1 cm using a stadiometer. Weight was measured to the nearest 0.1 kg using a calibrated digital scale. Body mass index (BMI) was calculated as weight (kg) divided by height squared (m²). Overweight and obesity were defined by the Indian Academy of Pediatrics (IAP) 2015 BMI growth charts [9]. Thinness was defined as BMI below the 3rd percentile, and normal nutritional status as BMI between the 3rd and 71st percentiles for boys and 3rd and 75th percentiles for girls according to IAP 2015 charts [9]. For international comparison, thinness was also classified using International Obesity Task Force (IOTF) cut-offs [10]. An adult-equivalent BMI of 23 kg/m² was used to define overweight, and 27 kg/m² to define obesity, as per IAP recommendations for Indian children [9,10].

**Figure 1:**
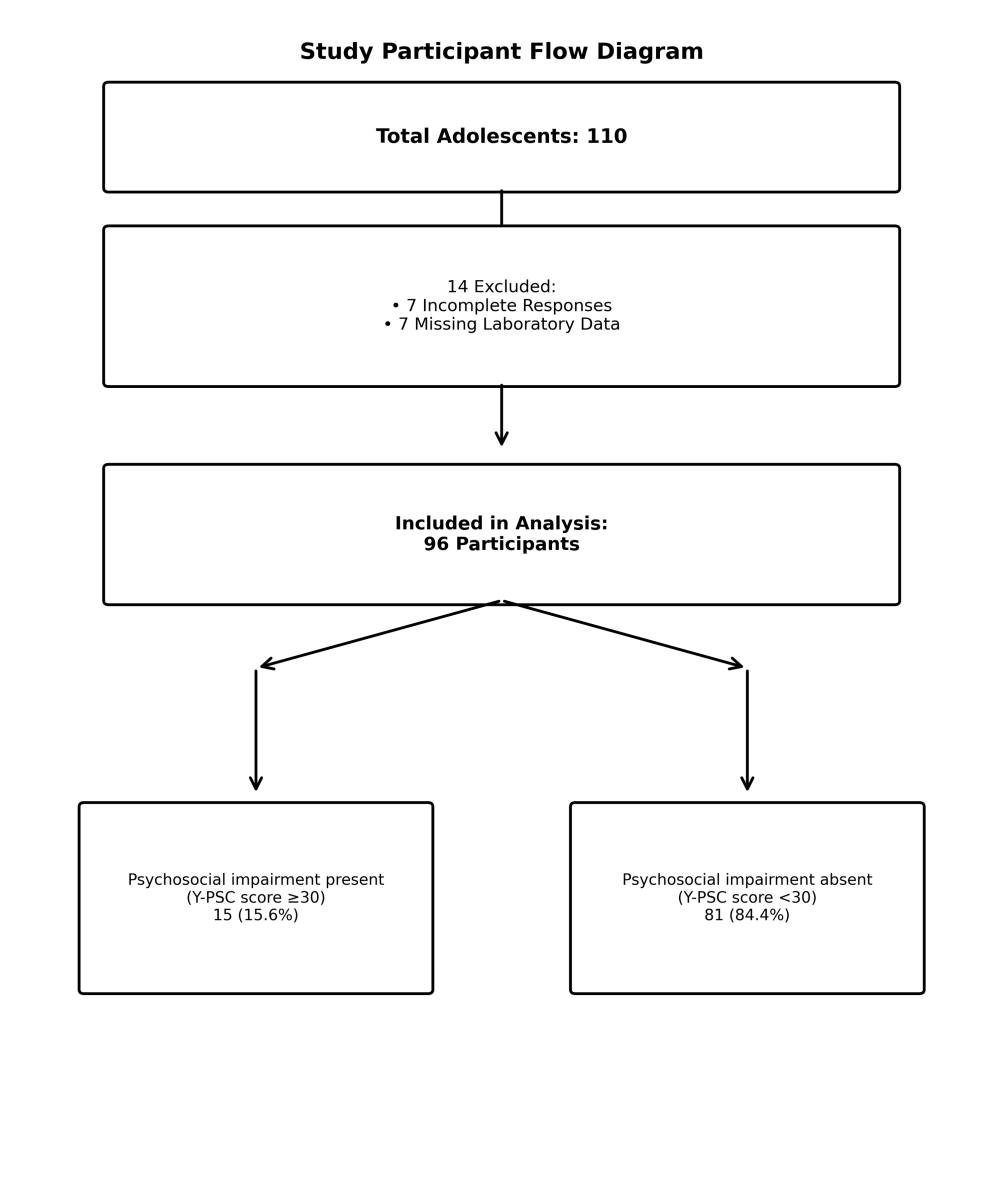
Flow of the study Y-PSC, Youth Pediatric Symptom Checklist

Psychosocial functioning was evaluated using the Y-PSC, a validated self-report screening instrument for adolescents aged 11 years and older. The Pediatric Symptom Checklist (PSC) was developed in the mid-1980s by Michael S. Jellinek, J. Michael Murphy, and colleagues as a brief, feasible screening tool for identifying psychosocial impairment in pediatric primary care and has demonstrated good reliability and validity across diverse child populations [11-14]. To address the limitations of exclusive parent reporting, particularly for internalizing symptoms, a youth self-report version (Y-PSC/PSC-Y) was subsequently developed [15]. The Y-PSC has shown satisfactory psychometric properties and criterion validity, supporting its use for screening psychosocial impairment in children and adolescents in clinical and community settings. The Y-PSC comprises 35 items reflecting internalizing, externalizing, and attention-related symptoms, scored on a 3-point Likert scale: "Never" (0), "Sometimes" (1), or "Often" (2). Total scores range from 0-70, with scores ≥30 indicating psychosocial impairment. Questionnaires with four or more unanswered items were considered invalid. The Y-PSC was administered in both English and Hindi following linguistic validation, demonstrating acceptable internal consistency (Cronbach's α=0.71). It is important to note that the Y-PSC is a screening tool designed to identify adolescents who may benefit from further mental health evaluation, not a diagnostic instrument. A score ≥30 indicates the need for a comprehensive psychiatric assessment but does not constitute a clinical diagnosis of any specific mental health disorder. This distinction is critical for the appropriate interpretation of our findings. Figure 1 shows the flow of the study.

Following psychosocial screening, 10 mL venous blood samples were collected aseptically under standardized conditions. Samples were collected in appropriate vacutainer tubes (EDTA (ethylenediaminetetraacetic acid) tubes for complete blood count, serum separator tubes for biochemical analyses) and transported to the laboratory in a cool box. All samples were processed within two hours of collection. Serum was separated by centrifugation at 3000 rpm for 10 minutes and stored at 2-8°C. All analyses were completed within 24 hours of sample collection to ensure analyte stability, particularly for vitamin B12 and folate.

Hemoglobin was measured using an automated hematology analyzer; serum ferritin using chemiluminescent microparticle immunoassay (CMIA); vitamin B12 using a CMIA-based intrinsic factor assay; serum folic acid using folate-binding protein CMIA; vitamin D using 25-hydroxyvitamin D CMIA; and total serum calcium using a photometric colorimetric assay.

To define micronutrient deficiencies within the study population, specific criteria were adapted for each parameter. Vitamin B12 deficiency was identified as serum B12 levels below 203 pg/mL (150 pmol/L), while folic acid deficiency was defined as serum folate below 3.0 ng/mL [16]. Anemia was classified by age- and sex-specific hemoglobin thresholds: less than 11.5 g/dL for children aged 5-11 years, less than 12.0 g/dL for those aged 12-14 years, less than 12.0 g/dL for girls above 15 years, and less than 13.0 g/dL for boys above 15 years [17]. Iron deficiency was determined by serum ferritin levels less than 15 ng/mL in the general healthy population [17]. For vitamin D, status was classified based on 25-hydroxyvitamin D levels: sufficient if above 50 nmol/L (>20 ng/mL), insufficient between 30 and 50 nmol/L (12-20 ng/mL), and deficient at less than 30 nmol/L (<12 ng/mL) [18].

Statistical analysis

Data were analyzed using IBM SPSS Statistics version 26.0 (IBM Corp., Armonk, USA). Continuous variables were assessed for normality; normally distributed data were summarized as mean±SD and compared using independent t-tests, while skewed data (e.g., ferritin) were presented as median (IQR) and analyzed using Mann-Whitney U tests. Categorical variables were compared using chi-square or Fisher's exact tests. Effect sizes were calculated using Cohen's d. Binary logistic regression identified factors independently associated with psychosocial impairment, with results reported as adjusted OR and 95% CI. Model diagnostics included assessment of multicollinearity (VIF), linearity of logit, influential observations, and model fit (Hosmer-Lemeshow test). Given the low events-per-variable ratio (EPV=2.5), bootstrap validation (1,000 iterations) and sensitivity analyses with a reduced model were performed. Statistical significance was set at P<0.05 (two-tailed).

## Results

Out of 110 adolescents who completed the questionnaire, 14 were excluded (seven for incomplete responses, seven for missing laboratory data), resulting in 96 participants for the final analysis. The mean age of participants was 13.36±1.48 years (range: 11-18 years), with a male-to-female ratio of 1.15:1 (52 males, 44 females). Table 1 presents the baseline demographic and clinical characteristics of the study population. Micronutrient deficiencies observed were vitamin D (94.8%), vitamin B12 (27.1%), serum folate (22.9%), ferritin (12.5%), and calcium (12.5%) (Table 2). The near-universal prevalence of vitamin D deficiency (94.8%) rendered this variable non-discriminatory for comparative analyses between groups with and without psychosocial impairment.

**Table 1:** Sociodemographic parameters of study participants (N=96) Y-PSC, Youth Pediatric Symptom Checklist; BMI, body mass index; -, not applicable

Variable (N=96)	Mean±SD	Median (IQR)/min-max	Frequency (percentage), N (%)
Age (years)	13.36±1.48	14.00 (12.00-14.00)/11-17	-
Gender	-	-	
• Female	-	-	44 (45.8%)
• Male	-	-	52 (54.1%)
Nutritional status (BMI for age)			
• Thinness	-	-	65 (67.7%)
• Normal	-	-	25 (26.0%)
• Overweight	-	-	6 (6.2%)
BMI (kg/m²)	17.72±3.43	17.40 (15.13-19.98)	-
Physical activity level (WHO)			
• Sedentary	-	-	51 (53.1%)
• Moderate	-	-	36 (37.5%)
• Vigorous	-	-	9 (9.4%)
Y-PSC score	18.16±9.18	18.00 (12.00-24.00)	-
Y-PSC score ≥30	-	-	15 (15.6%)

**Table 2:** Micronutrient status and prevalence of deficiencies (N=96) *Anemia. **Median (IQR) reported for ferritin due to skewed distribution.

Parameter	Mean±SD	Median (IQR)	Deficiency, n (%)
Hemoglobin (g/dL)	11.66±1.56	11.80 (10.85-12.55)	38 (39.6%)*
Serum ferritin (ng/mL)**	42.0 (23.0-72.5)	49.47 (26.89-84.38)	12 (12.5%)
Serum vitamin B12 (pg/mL)	271.5±195.8	242.00 (189.50-320.00)	26 (27.1%)
Serum folate (ng/mL)	8.4±4.2	6.0 (4.2-8.5)	22 (22.9%)
25-hydroxyvitamin D (ng/mL)	9.8±4.1	7.90 (4.80-12.15)	91 (94.8%)
Serum calcium (mg/dL)	9.2±0.6	9.47 (8.99-9.77)	12 (12.5%)

Psychosocial impairment (Y-PSC score ≥30) was identified in 15.6% (15/96) of participants. Adolescents with psychosocial impairment demonstrated significantly lower mean serum vitamin B12 levels compared to those without impairment (191.86±63.00 vs. 291.09±207.02 pg/mL; P=0.039), representing a mean difference of -99.23 pg/mL (95% CI: -154.45 to -44.01) with a moderate effect size (Cohen's d=0.52). Vitamin B12 deficiency was significantly more prevalent among adolescents with psychosocial impairment (80.0% vs. 23.4%; P<0.001). Median serum ferritin levels were significantly lower in adolescents with psychosocial impairment (28.50 ng/mL, IQR: 21.00-42.00) compared to those without impairment (45.00 ng/mL, IQR: 24.00-78.00; Mann-Whitney U test, P=0.041). The use of nonparametric testing was necessitated by the markedly skewed distribution of ferritin values, particularly in the nonimpaired group (Shapiro-Wilk test P<0.001). No statistically significant differences were observed between groups for other biochemical parameters, including folic acid, vitamin D, calcium, and hemoglobin levels (P>0.05) (Table 3 and Table 4 [18]). Table 5 presents the unadjusted (bivariate) associations between potential predictor variables and psychosocial impairment. In unadjusted analyses, vitamin B12 deficiency showed a strong association with psychosocial impairment (OR=13.11; 95% CI: 3.35-51.28; P<0.001). When analyzed as continuous variables, both lower serum vitamin B12 levels (OR=0.95 per 10 pg/mL increase; 95% CI: 0.91-0.99; P=0.015) and lower serum ferritin levels (OR=0.89 per 10 ng/mL increase; 95% CI: 0.80-0.99; P=0.028) were significantly associated with increased odds of psychosocial impairment. Multiple logistic regression analysis revealed serum vitamin B12 levels as significantly associated with psychosocial impairment (OR=0.985; 95% CI: 0.972-0.997; P=0.017), indicating that higher vitamin B12 levels were independently associated with reduced odds of psychosocial impairment. Serum ferritin levels showed a trend toward significance (OR=0.976; 95% CI: 0.948-1.004; P=0.098) (Table 6). Serum ferritin levels were not significantly associated with psychosocial impairment in the adjusted model (OR=0.976; 95% CI: 0.948-1.004; P=0.098), despite showing a significant association in unadjusted analyses. This model includes six predictor variables with only 15 events (Y-PSC ≥30), yielding an EPV ratio of 2.5, substantially below the recommended minimum of 10. This low EPV can result in overfitting, unstable coefficient estimates, and overly optimistic confidence intervals. Results should be interpreted as exploratory and hypothesis-generating only.

**Table 3:** Comparison of sociodemographic, nutritional, and biochemical parameters of participants (n=96) based on psychosocial score categories Pearson’s chi-square test was used when all expected cell frequencies were ≥5; Fisher’s exact test was used when any expected cell frequency was <5. **p<0.001 (statistically significant) ^†^Expected cell frequency <5, requiring Fisher’s exact test; χ², Chi-square statistic; df, degrees of freedom; -, not applicable; PSC, Pediatric Symptom Checklist; BMI, body mass index

Variable (N=96)	PSC <30 (n=81), N (%)	PSC ≥30 (n=15), N (%)	Test type	χ²	df	p-value
Age group			Pearson χ²	0.09	1	0.765
11-13 years	39 (48.15%)	6 (40.00%)				
14-18 years	42 (51.85%)	9 (60.00%)				
Gender			Pearson χ²	0.68	1	0.408
Female	36 (44.44%)	9 (60.00%)				
Male	45 (55.56%)	6 (40.00%)				
BMI category			Fisher’s exact	-	-	0.620
Thinness	57 (70.35%)	9 (60.00%)				
Normal	19 (23.46%)	5 (33.33%)				
Overweight and obese	5 (6.17%)	1 (6.67%)				
Hemoglobin status			Pearson χ²	0.05	1	0.816
Anemia	32 (39.51%)	7 (46.67%)				
Normal	49 (60.49%)	8 (53.33%)				
Vitamin B12 status			Fisher’s exact^†^	-	-	<0.001**
Deficient	19 (23.46%)	12 (80.00%)				
Normal	62 (76.54%)	3 (20.00%)				
Folic acid status			Fisher’s exact^†^	-	-	0.508
Deficient	20 (24.69%)	2 (13.33%)				
Normal	61 (75.31%)	13 (86.67%)				
Serum calcium status			Fisher’s exact	-	-	0.203
Deficient	12 (14.81%)	0 (0.00%)				
Normal	69 (85.19%)	15 (100.00%)				
Vitamin D status			Fisher’s exact^†^	-	-	1.000
Deficient	75 (92.59%)	14 (93.33%)				
Normal	6 (7.41%)	1 (6.67%)				
Ferritin status			Fisher’s exact^†^	-	-	1.000
Deficient	11 (13.58%)	2 (13.33%)				
Normal	70 (86.42%)	13 (86.67%)				
Physical activity level			Fisher’s exact	-	-	0.520
Moderate	32 (39.51%)	4 (26.67%)				
Sedentary	41 (50.62%)	10 (66.67%)				
Vigorous	8 (9.88%)	1 (6.67%)				
Diet type			Fisher’s exact^†^	-	-	0.545
Nonvegetarian	57 (70.37%)	9 (60.00%)				
Vegetarian	24 (29.63%)	6 (40.00%)				

**Table 4:** Comparison of mean nutritional and biochemical parameters of participants (n=96) by psychosocial score category Values are expressed as mean±SD. Comparisons between the PSC <30 and PSC ≥30 groups were performed using the independent samples t-test. *p<0.05 indicates statistical significance. **Ferritin values are expressed as median (IQR) due to skewed distribution (Shapiro-Wilk test P<0.001) and compared using the Mann-Whitney U test. ^†^Hemoglobin cut-offs (WHO): <11.5 g/dL (5-11 y), <12.0 g/dL (12-14 y), <12.0 g/dL (females ≥15 y), <13.0 g/dL (males ≥15 y). PSC, Pediatric Symptom Checklist; BMI, body mass index; IAP, Academy of Pediatrics; IOTF, International Obesity Task Force

Parameter	Reference range/cut-off (guideline-based)	PSC <30 (n=81), mean±SD	PSC ≥30 (n=15), mean±SD	Test statistic (t-value)	p-value
BMI (kg/m²)	Age- and sex-specific percentiles (IAP 2015; IOTF-based)	17.58±3.43	18.24±3.49	0.66	0.513
Vitamin B12 (pg/mL)	≥203 pg/mL (150 pmol/L)	291.09±207.02	191.86±63.00	2.10	0.039*
Folate	Serum folate ≥4 ng/mL (10 nmol/L)	5.30±1.82	5.85±2.79	0.98	0.332
Serum calcium (mg/dL)	8.5-010.5 mg/dL	9.27±0.78	9.62±0.60	1.65	0.102
25-hydroxyvitamin D (ng/mL)	Sufficient >20; insufficient 12-20; deficient <12	14.71±13.91	27.21±65.71	1.16	0.250
Ferritin** (ng/mL)	≥15 ng/mL (healthy population)	45.00 (24.00-78.00)	28.50 (21.00-42.00)	U=404**	<0.041*
Hemoglobin (g/dL)	WHO age- and sex-specific cut-offs^†^	11.64±1.58	11.57±1.34	0.17	0.865

**Table 5:** Unadjusted associations between nutritional parameters and psychosocial impairment *Ferritin was analyzed using log-transformed values in logistic regression due to skewed distribution. OR, odds ratios

Variable (N=15)	Unadjusted OR (95% CI)	P-value
Vitamin B12 deficiency (<203 pg/mL)	13.11 (3.35-51.28)	<0.001
Iron deficiency (ferritin <15 ng/mL)	1.99 (0.48-8.28)	0.343
Folate deficiency (<3.0 ng/mL)	1.89 (0.57-6.28)	0.297
Anemia	1.94 (0.65-5.82)	0.237
Hypocalcemia	1.09 (0.22-5.45)	0.916
Serum vitamin B12 (per 10 pg/mL increase)	0.95 (0.91-0.99)	0.015
Serum ferritin (per 10 ng/mL increase)*	0.89 (0.80-0.99)	0.028
Serum folate (per ng/mL increase)	0.96 (0.84-1.09)	0.539
Hemoglobin (per g/dL increase)	0.82 (0.55-1.22)	0.327
25-hydroxyvitamin D (per ng/mL increase)	0.96 (0.85-1.09)	0.54
Total calcium (per mg/dL increase)	0.88 (0.39-1.98)	0.753

**Table 6:** Multivariable adjusted logistic regression model for predictors of psychosocial impairment *A p-value <0.05 was considered statistically significant. Multiple logistic regression was performed to identify independent predictors of psychosocial impairment. ORs with 95% CI were calculated as Exp(B). OR, odds ratios; χ², Chi-square statistic; df, degrees of freedom; SE, standard error

Variable (N=15)	β (B)	SE	Wald χ²	df	p-value	OR (Exp B)	95% CI for OR
Vitamin B12 (pg/mL)	−0.015	0.006	5.727	1	0.017*	0.985	0.972-0.997
Folic acid (ng/mL)	0.141	0.179	0.621	1	0.431	1.151	0.811-1.635
Serum calcium (mg/dL)	0.380	0.558	0.463	1	0.496	1.462	0.490-4.367
Vitamin D (ng/mL)	0.000	0.018	0.001	1	0.980	1.000	0.965-1.035
Ferritin (ng/mL)	-0.024	0.015	2.735	1	0.098	0.976	0.948-1.004
Hemoglobin (g/dL)	0.100	0.309	0.105	1	0.746	1.106	0.603-2.027

In the multivariable adjusted model, serum vitamin B12 levels were significantly associated with psychosocial impairment (OR=0.985; 95% CI: 0.972-0.997; P=0.017), indicating that higher vitamin B12 levels were independently associated with reduced odds of psychosocial impairment. Each 1 pg/mL increase in serum vitamin B12 was associated with a 1.5% reduction in the odds of psychosocial impairment after adjusting for other variables. Serum ferritin levels showed a trend toward significance but were not significantly associated with psychosocial impairment in the adjusted model (OR=0.976; 95% CI: 0.948-1.004; P=0.098), despite showing a significant association in unadjusted analyses (Table 4). This was not confirmed in multivariable models, possibly due to confounding (notably by vitamin B12), limited sample size, collinearity, or unmeasured factors such as inflammation. A reduced logistic regression with only vitamin B12 and ferritin (EPV=7.5) found that vitamin B12 remained significantly associated with psychosocial impairment (OR=0.983; 95% CI: 0.971-0.995; P=0.006), while ferritin showed a nonsignificant trend (OR=0.972; 95% CI: 0.943-1.002; P=0.067). Bootstrap validation (1000 iterations) produced similar estimates but with wider confidence intervals, highlighting some instability due to the small sample size.

## Discussion

This pilot study examined associations between micronutrient deficiencies and psychosocial impairment among 96 Indian adolescents attending a tertiary care hospital in Delhi. Psychosocial impairment in 15.6% (15/96) of the adolescent cohort aligns with existing literature from similar populations, though variations exist across different settings and methodologies [19,20]. These findings are particularly significant within the Indian healthcare context, where adolescent mental health and nutrition represent intersecting public health challenges. The Comprehensive National Nutrition Survey (CNNS) 2016-18 reported vitamin B12 deficiency in 30.9% and folate deficiency in 35.6% of Indian adolescents nationally [21,22]. In this study, vitamin B12 deficiency was found in 26 (27.1%) adolescents, which aligns closely with national data, while a striking 12/15 (80%) of psychosocially impaired adolescents were vitamin B12 deficient, representing a concentrated risk population. However, this association does not establish causality, and the direction of the relationship remains unclear from this cross-sectional design.

The study observed a statistically significant association between vitamin B12 deficiency and psychosocial impairment, with 80% of affected adolescents showing vitamin B12 deficiency compared to 23.4% in the unaffected group. This association remained significant in multivariable analysis, though the clinical significance of the effect size warrants careful consideration. The observed OR of 0.985 per 1 pg/mL increase in vitamin B12 translates to an OR of approximately 0.86 for a 10 pg/mL increase, or 0.14 for a 100 pg/mL increase. While statistically significant, the clinical meaningfulness of this effect size is uncertain. The difference in mean vitamin B12 levels between groups was approximately 100 pg/mL (192 vs. 291 pg/mL), which would correspond to an approximately 86% reduction in odds of psychosocial impairment. However, given the wide confidence interval and the EPV limitation, this estimate should be interpreted cautiously. The mechanistic basis for this association involves vitamin B12's critical role in one-carbon metabolism and neurotransmitter synthesis during the crucial period of adolescent brain development [23,24]. Vitamin B12 deficiency impairs the remethylation of homocysteine to methionine, disrupting S-adenosylmethionine production, a key methyl donor for dopamine, serotonin, and norepinephrine synthesis [25]. During adolescence, when neural circuits underlying emotional regulation and executive function are rapidly maturing, adequate vitamin B12 availability may be particularly crucial for optimal neurotransmitter balance and psychological well-being.

The predominance of vegetarian diets in many Indian regions may contribute substantially to vitamin B12 deficiency, as plant-based foods generally lack bioavailable vitamin B12 [25]. In our sample, 60.4% of adolescents followed a vegetarian diet, though the association between a vegetarian diet and psychosocial impairment was not statistically significant (P=0.588). This dietary pattern necessitates attention to vitamin B12 supplementation or fortified food consumption.

Adolescents with psychosocial impairment showed significantly lower serum ferritin levels in unadjusted analysis (median 28.5 vs. 45.0 ng/mL, P=0.04), consistent with research on iron's importance in neurocognitive function [26,27]. Iron deficiency affects brain myelination, monoamine metabolism, and neurotransmitter balance, potentially contributing to attention deficits, mood disturbances, and behavioral problems during this critical developmental period [28,29]. The association between ferritin and psychosocial impairment was not statistically significant in the adjusted model (P=0.098), despite significance in the unadjusted analysis. This discordance may reflect confounding by vitamin B12 or other covariates, limited statistical power, and potential collinearity between ferritin and other predictors. Notably, these associations occurred independently of hemoglobin status, highlighting significant limitations of using hemoglobin alone as a nutritional screening tool in adolescent healthcare. This finding suggests that subclinical iron deficiency may impact mental health and cognitive function before overt anemia develops, supporting the need for more comprehensive micronutrient assessment in adolescent health screening protocols.

No significant associations were observed between psychosocial impairment and serum folate, vitamin D, or calcium levels. The lack of association with vitamin D is likely explained by the near-universal deficiency (94.8%) in our sample, which precluded meaningful between-group comparisons. This extremely high prevalence of vitamin D deficiency is consistent with other Indian studies and reflects limited sun exposure, cultural practices (clothing coverage), and dietary factors. However, it renders vitamin D noninformative as a discriminatory variable in this study.

Our findings complement emerging evidence from other Indian studies examining nutrition-mental health associations in adolescents. Previous research has reported associations between micronutrient intake and psychological outcomes in Indian adolescent populations, while other studies have demonstrated relationships between multiple micronutrient deficiencies and cognitive performance in urban Indian schoolchildren [30,31]. However, this study uniquely used the validated Y-PSC screening tool, providing more clinically relevant insights for pediatric practice. However, several important differences should be noted. Our study was hospital-based, whereas many previous studies were community- or school-based, limiting direct comparability and introducing potential selection bias. Additionally, the markedly higher prevalence of thinness in our sample (67.7% vs. ~20-30% in CNNS) and the cross-sectional design further restrict generalizability and preclude causal inference.

If confirmed in larger, adequately powered prospective studies, these findings could have important clinical and public health implications. Current screening protocols primarily focus on anthropometric measurements and hemoglobin levels, potentially missing subclinical micronutrient deficiencies that significantly impact mental health outcomes. A targeted screening approach incorporating serum vitamin B12 and ferritin levels in adolescents presenting with behavioral concerns, academic difficulties, or psychosocial symptoms could identify modifiable risk factors amenable to cost-effective interventions.

Given resource constraints in Indian healthcare settings, selective screening of high-risk adolescents may be more feasible than universal micronutrient testing. Risk factors for targeted screening could include psychosocial symptoms, predominantly vegetarian diets, poor academic performance, recurrent infections, or family history of mental health disorders. The relatively low cost of vitamin B12 and iron supplementation compared to long-term mental health interventions supports the economic rationale for such screening approaches; however, further large-scale studies are required to establish causality. If the results are replicated, integration of micronutrient assessment into existing programs could enhance early identification and intervention capabilities. School-based screening programs, already established for vision and dental health in many Indian states, could potentially incorporate simple psychosocial screening tools like the Y-PSC alongside targeted micronutrient testing for identified at-risk adolescents.

For practical implementation in Indian healthcare settings, a tiered screening approach could be considered: primary screening using validated psychosocial tools (Y-PSC) in school or clinic settings, followed by targeted micronutrient assessment for adolescents with elevated scores. This approach would optimize resource utilization while identifying adolescents most likely to benefit from nutritional interventions. However, all these implications are contingent on confirmation of findings in larger, well-designed studies, including randomized controlled trials of micronutrient supplementation with mental health outcomes.

Limitations

Hospital-based recruitment and exclusion of admitted patients may introduce selection bias. The markedly higher prevalence of thinness in our sample (67.7% vs. 20-30% nationally) further limits generalizability to the broader adolescent population. The low events-per-variable ratio produces unstable, exploratory regression estimates. Additionally, the cross-sectional design precludes causal inference, the Y-PSC is a screening (not diagnostic) tool, and the single-center design further limits generalizability.

Strengths

Despite these limitations, the study has several strengths, including use of a validated, standardized screening tool (Y-PSC) with demonstrated psychometric properties, comprehensive assessment of multiple micronutrients using objective biomarkers, linguistic validation of the Y-PSC in Hindi for cultural appropriateness, standardized laboratory methods with quality control procedures, transparent reporting of limitations and appropriate caution in interpretation, contribution to the limited evidence base on nutrition-mental health associations in Indian adolescents, and pilot data to inform the design of larger, adequately powered future studies.

## Conclusions

This pilot study demonstrates preliminary associations between psychosocial impairment and deficiencies in vitamin B12 and ferritin among Indian adolescents attending a tertiary care hospital in Delhi. While causality cannot be established from this cross-sectional design, and the findings are limited by small sample size, selection bias, and methodological constraints (particularly the low events-per-variable ratio in multivariable analyses), the results suggest the potential value of incorporating targeted micronutrient screening into routine adolescent psychosocial assessments. If confirmed in larger, adequately powered prospective studies and randomized controlled trials, identification of modifiable nutritional factors linked to psychosocial impairment could provide a basis for developing cost-effective interventions to improve adolescent mental health outcomes in India and other low- and middle-income settings with high prevalence of micronutrient deficiencies. These preliminary findings provide hypothesis-generating data to inform the design of future confirmatory studies and, if validated, could eventually support integration of nutritional and mental health screening into adolescent healthcare and national health programs.
